# On-field low-frequency fatigue measurement after repeated drop jumps

**DOI:** 10.3389/fphys.2022.1039616

**Published:** 2022-11-09

**Authors:** Jade Ridard, Vianney Rozand, Guillaume Y. Millet, Thomas Lapole

**Affiliations:** ^1^ Laboratoire Interuniversitaire de Biologie de la Motricité, UJM Saint-Etienne, University Lyon, SAINT-ETIENNE, France; ^2^ Institut Universitaire de France (IUF), Paris, France

**Keywords:** drop jumps, eccentric contraction, fatigability, prolonged low-frequency force depression, athletic performance

## Abstract

**Purpose:** Monitoring fatigue is now commonly performed in athletes as it can directly impact performance and may further increase the risk of injury or overtraining syndrome. Among the exercise-induced peripheral alterations, low-frequency fatigue (LFF) assessment is commonly restricted to in-lab studies. Measuring LFF on-field would allow athletes and coaches to assess muscle fatigability on a regular basis. The aim of the present study was therefore to validate a new portable device allowing quadriceps LFF assessment in the field.

**Methods:** LFF was assessed in 15 active and healthy participants before (PRE) and after (POST) a series of drop jumps. LFF was assessed, thanks to a dedicated device recording evoked force to muscle submaximal electrical low- and high-frequency stimulation. Changes in the low- to high-frequency force ratio (further referred to as Powerdex^®^ value) were compared to the changes in the ratio of evoked force induced by paired-pulse femoral nerve electrical stimulation at 10 and 100 Hz (i.e., DB10/DB100 ratio). Maximal voluntary contraction (MVC) and voluntary activation (VA) were also measured.

**Results:** MVC decreased (*p* < 0.001), whereas VA was not affected by the fatiguing task (*p* = 0.14). There was a decrease in the DB10/DB100 ratio (from 96.4% to 67.3%, *p* < 0.001) as well as in the Powerdex value (from 74.0% to 55.7%, *p* < 0.001). There was no significant difference between POST values (expressed in percentage of PRE values) of the DB10/DB100 ratio and Powerdex (*p* = 0.44), and there was a significant correlation between the changes in Powerdex^®^ and DB10/DB100 (r = 0.82, *p* < 0.001).

**Conclusion:** The on-field device we tested is a valid tool to assess LFF after a strenuous exercise consisting of repeated drop jumps as it evidences the presence of LFF similarly to a lab technique. Such a device can be used to monitor muscle fatigability related to excitation–contraction in athletes.

## Introduction

Athletes, especially elite athletes, are exposed to high training and competition loads, making the monitoring of fatigue extremely relevant (Thorpe et al., 2017). As defined by Enoka and Duchateau (2016), fatigue can be conceptualized as a disabling symptom in which physical and cognitive functions are limited by interactions between performance fatigability (i.e., the decline in an objective measure of performance) and perceived fatigability (i.e., changes in the sensations that regulate the integrity of the performer). Fatigue directly impacts sport performance, and may further increase the risk of injury or induce an overtraining syndrome in case of prolonged excessive imbalance between training/competition loads and recovery periods (Thorpe et al., 2017; Cheng et al., 2020).

A reduction in maximal force during an isometric maximal voluntary contraction (MVC) is thought to provide the most straightforward evidence of performance fatigability (Carroll et al., 2017). Part of the alterations within the neuromuscular system can occur at the central nervous system level, as evidenced by a decreased voluntary activation level (VA) assessed through the superimposed twitch technique (Merton, 1954). Alterations can also occur at the peripheral level through impaired muscle contractility (Allen et al., 2008), which can be assessed through peripheral nerve or muscle electrical stimulation (Millet et al., 2011). As such, performance fatigability can be ultimately defined to occur when the force output is lower than what is expected for a given voluntary or evoked stimulus (MacIntosh and Rassier, 2002). In other words, despite maximal voluntary contraction being preserved, performance fatigability may still exist if the force induced by certain types of stimulation is depreciated.

Among the exercise-induced peripheral alterations, low-frequency fatigue (LFF), also known as prolonged low-frequency force depression (Allen et al., 2008), is a long-lasting form of muscle fatigability and is characterized by a larger decrease of force at low stimulation frequencies than at high stimulation frequencies (Edwards et al., 1977; Jones, 1996). LFF is suggested to reflect excitation–contraction coupling failure through decreased Ca^2+^ release within muscle fibers (Edwards et al., 1977; Hill et al., 2001; Keeton and Binder-Macleod, 2006; Dargeviciute et al., 2013). LFF is notably believed to be a primary source of peripheral alterations after eccentric contractions (Martin et al., 2004; Iguchi and Shields, 2010; Skurvydas et al., 2016; Kamandulis et al., 2019). But LFF and/or alteration of Ca^2+^ release has been observed following other types of fatiguing tasks such as intense exercise (Lattier et al., 2004; Skurvydas et al., 2016) or exercise inducing glycogen depletion (ørtenblad et al., 2011).

The gold standard to assess LFF is the ratio of low- to high-frequency force responses to trains of peripheral nerve electrical stimulation at supramaximal intensity (Allen et al., 2008). Due to the discomfort induced by such tetanic nerve stimulation, evoked forces to paired stimuli at 10Hz and 100 Hz have been proposed as an alternative method (Verges et al., 2009). But because of the complexity of this kind of measurements (i.e., electrode placement, specific material, and discomfort), LFF assessments are commonly restricted to in-lab studies. Measuring LFF on-field would however provide great insight to athletes and coaches on athletes’ muscle fatigability, allowing better management of training/competition loads. This is the goal of Myocene^®^, a new portable device allowing quadriceps LFF assessment on the field. For that purpose, it is composed of an easy-to-transport knee extensor dynamometer integrating an electrical stimulator for muscle transcutaneous stimulation. Considering that the relative decrease in the low- to high-frequency ratio after a fatiguing task is not different whether supramaximal nerve stimulation or submaximal muscle stimulation is applied (Martin et al., 2004), the device uses trains of submaximal stimuli applied to the quadriceps muscle to reduce discomfort and simplify its acceptability by athletes. But the outcome provided by this on-field device (i.e., the so-called Powerdex) remains to be validated when compared to laboratory measurements.

The aim of this study was to validate the use of a portable on-field device to measure LFF induced by a series of drop jumps, that is, exercise consisting of stretch-shortening cycles with a strong eccentric component. We therefore compared the exercise-induced decrease in Powerdex and the decrease in the ratio of 10- to 100-Hz doublets in an in-lab setting. We hypothesized that the magnitude of LFF would be correlated between both methods.

## Methods

### Participants

Fifteen active and healthy participants (11 men and four women; age: 26 ± 5 yr; body weight: 70 ± 10 kg; height: 174 ± 9 cm) participated in the present experiment. They self-reported their main strength as either explosive (n = 9) or enduring (n = 6). They reported no history of neurological or musculoskeletal impairment. Participants were asked to refrain from strenuous and unaccustomed physical activity for 48 h before testing to minimize the risk of prior fatigue or muscle damage. The study protocol was approved by the local Ethics Committee and was in accordance with the latest update of the Helsinki Declaration (except for registration in a database). All subjects gave their written informed consent before participation.

### Study design

**FIGURE 1:**
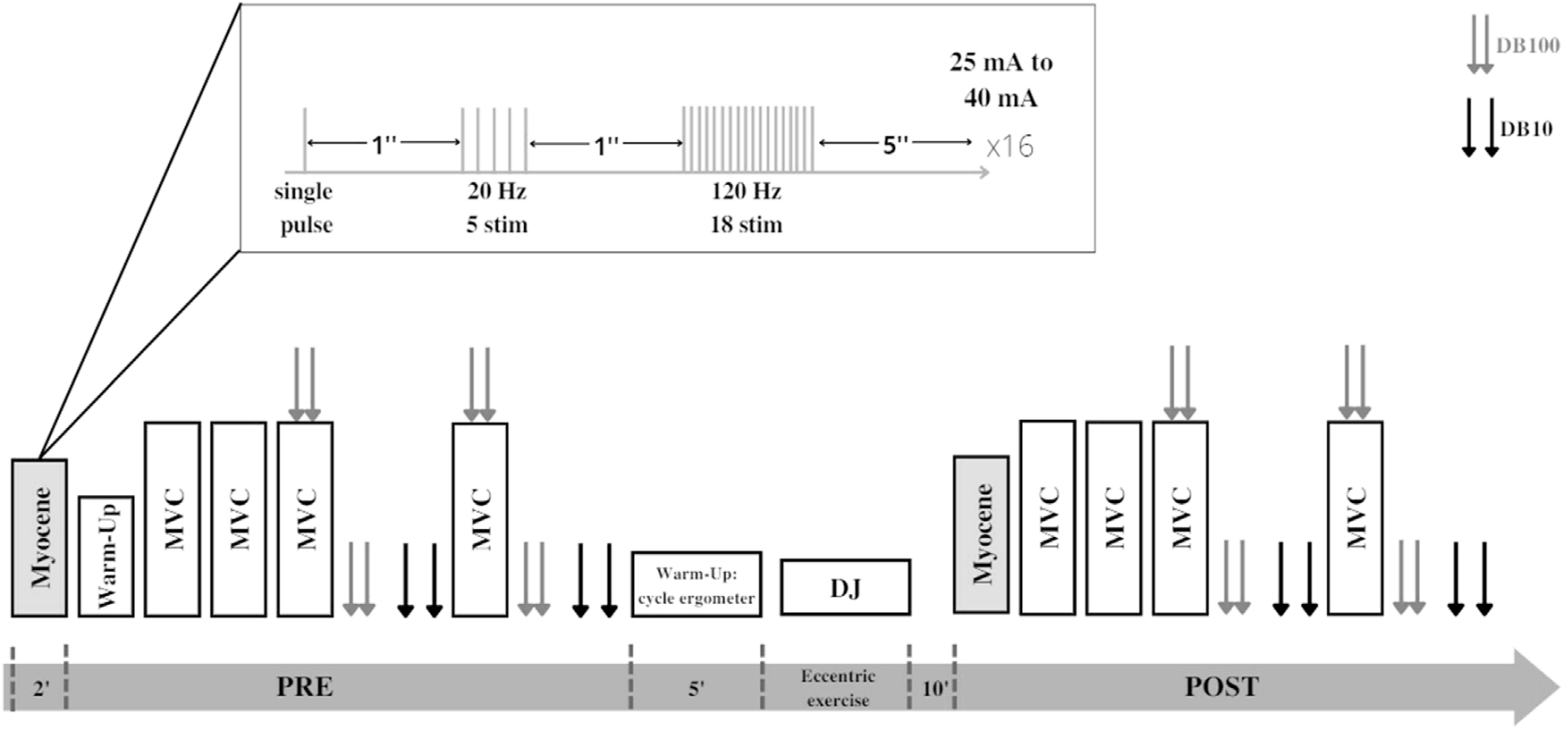
Experimental design of the study. Each participant performed the Myocene^®^ measurement (single pulse + low-frequency train + high-frequency train) and maximal voluntary contractions (MVCs) with peripheral nerve stimulation performed before (PRE) and after (POST) series of drop jumps (DJs). Peripheral nerve stimulations are represented by double gray (paired stimulations at 100 Hz, DB100) and double black arrows (paired stimulations at 10 Hz, DB10).

Participants visited the laboratory on two occasions. During the first session, participants were familiarized with the experimental procedures. At least seven days later, participants went back to the laboratory for the evaluation session. This first evaluation consisted of quadriceps LFF assessment (PRE) using the on-field device. Then, participants moved to the laboratory dynamometer for a neuromuscular function evaluation: maximal voluntary contraction (MVC), maximal voluntary activation level (VA), and evoked responses to 10- and 100-Hz doublet on relaxed muscle. Participants were then asked to perform an intense eccentric exercise composed of repetitive drop jumps (DJs). Participants were then retested (POST) in the same order as in PRE. A 10-min resting period was observed between the last DJ and POST measurements to avoid the neuromuscular evaluation to be affected by metabolic fatigue. All measurements were performed on the right leg. The study design is described in Figure 1.

### LFF assessment using the on-field device

**FIGURE 2:**
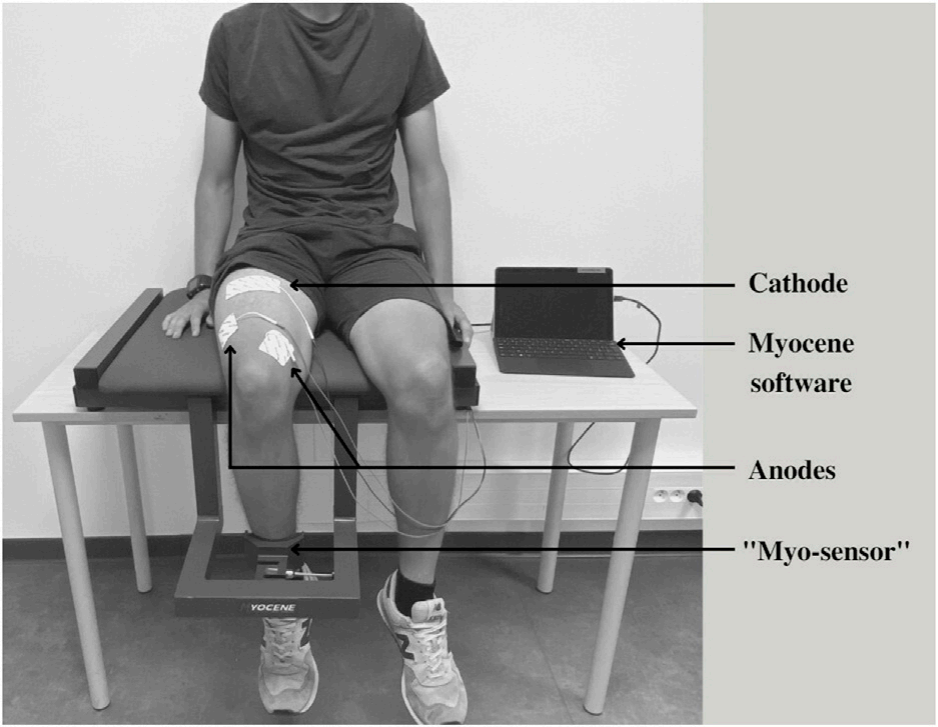
Illustration of the Myocene^®^ device. The “Myo-sensor” records the evoked force for each stimulation sent through the electrodes connected to the Myocene^®^: cathode placed transversely across the width of the proximal portion of the quadriceps femoris; anodes placed over the vastus lateralis and vastus medialis muscles. Evoked force data are processed, thanks to the Myocene^®^ software to provide the Powerdex value.

This study was performed using the on-field Myocene^®^ device (Figure 2). Participants sat on the seating of the device with their leg in contact with the “Myo-sensor,” that is, a dedicated force sensor recording evoked forces at a rate of 4 kHz. Muscle electrical stimulations (biphasic square wave with a pulse width of 400 µs) were applied using three electrodes (MyoPro-1-electrode, Myocene, Liège, Belgium). The cathode (5 × 10 cm) was placed transversely across the width of the proximal portion of the quadriceps femoris, and the anodes (5 × 5 cm) were, respectively, placed over the vastus lateralis and the vastus medialis muscles. Pre-programmed electrical stimuli trains were directly sent by the device that was driven by the Myocene^®^ software. The series of stimuli consisted of sets including 1) a single pulse, 2) a train of 5 stimuli at low frequency (20 Hz), and 3) a train of 18 stimuli at high frequency (120 Hz). One second separated each stimulation (Figure 1). In total, 16 sets were performed, with 5 s in-between, and the intensity of stimulation was progressively increased by steps of 1 mA at each set (from 25 mA to 40 mA). The total duration of LFF assessment using the device was 2 min. The Myocene^®^ software integrates a specific algorithm allowing the instantaneous and automatic measurement of LFF. Within each set, the ratio of low- to high-frequency evoked forces was calculated, and the median value of all ratios was provided as an outcome, that is, the so-called Powerdex. This Powerdex value was therefore retained for analysis in the present study.

### Quadriceps neuromuscular function evaluation

Participants sat on a custom-built chair with knees and hip at 90° of flexion. Force was recorded using a calibrated load cell (Meiri F2732 200 daN, Celians, Montauban, France) fixed to the chair and attached above the ankle malleoli of participants *via* a non-compliant strap. Movements of the upper body were minimized using two belts across the thorax. The force signal was acquired using a PowerLab data acquisition system (16/30-ML880/P, ADInstruments, Bella Vista, Australia) at a sampling rate of 2 kHz. During all measurements, participants were provided with real-time feedback of their force traces on a screen. Strong verbal encouragements were given during MVCs.

Neuromuscular function evaluation (Figure 1) began with a free warm-up (only in PRE) consisting of submaximal contractions of the quadriceps. When the subjects felt warm enough, they had to perform four 3-s MVCs with 1 min of rest in-between. The first two MVCs were performed without any stimulation. For the third and fourth MVCs, femoral nerve electrical stimulations were delivered during and after the MVCs to assess VA, muscle contractility, and LFF. Square-wave stimuli of 200 µs duration (400-V maximal output voltage) were delivered to the femoral nerve *via* a constant current stimulator (DS7R, Digitimer, Welwyn Garden City, UK). The 30-mm diameter surface cathode (Meditrace 100) was placed on the femoral triangle and the anode (5 × 10 cm Durastick Plus, DJO Global, Vista, CA) in the gluteal fold. Stimulus intensity was initially determined before baseline MVCs, starting at 30 mA and then increasing the intensity gradually until the maximum quadriceps twitch response was reached. A supramaximal intensity of 130% was then chosen to ascertain full spatial recruitment with small changes in the electrode position (Millet et al., 2011). Paired pulse 100-Hz electrical stimuli were superimposed onto the third and fourth MVCs when the force reached a plateau, eliciting a superimposed doublet (i.e., Db100_sup_). One hundred hertz doublets (DB100) were also delivered at rest 2 s after MVCs and ten-hertz doublets (DB10) were delivered 2 s later*.* The peak force among the four MVCs was retained for analysis. VA was calculated as follows (Strojnik and Komi, 1998):

$$VA=1-\frac{\left({Db100}_{sup}\right)\times \left(\frac{force\;at\;stimulation}{peak\;force}\right)}{Db100}\times 100.$$

The maximal VA value was retained for analysis. Finally, LFF was assessed using the mean DB10/DB100 ratio from the two measurements. All data were analyzed offline using LabChart 8 software (ADInstruments).

### Eccentric exercise

After a 5-min free warm-up on a cycle ergometer, participants performed repetitive DJs with hands on hips from a box 0.45 m high to 90° knee flexion, followed by an immediate maximal countermovement rebound. A 15-s interval separated DJs (Skurvydas et al., 2016; Kamandulis et al., 2019). The knee angle was visually monitored by the investigator, and appropriate feedback was given to participants when necessary. Because of the heterogeneous level of training of the participants, the number of DJs was individualized. More specifically, the Powerdex was calculated immediately after 100 DJs and then immediately after every 20 additional DJs. The DJs were stopped when the decrease in Powerdex reached a plateau that was visually determined.

### Statistical analysis

Results are presented as mean ± standard deviation. Normal distribution was checked using the Shapiro–Wilk test. When the assumption of normality was met, paired t-tests were performed to compare PRE and POST values (i.e., Powerdex, DB10/DB100, MVC). When normality was not met, a Wilcoxon test was performed (i.e., VA). A paired t-test was also performed between POST values of the DB10/DB100 ratio and Powerdex, and both expressed in percentage of PRE values. Effect size is presented as Cohen’s d with values representing small (d = 0.2), medium (d = 0.5), and large (d = 0.8) effects. The correlation between POST values (expressed in percentage of PRE values) of Powerdex and DB10/DB100 was evaluated using the Pearson correlation test (r). Differences were considered significant when *p* < 0.05. Statistical analyses were performed using JASP software (JASP 0.14.0.0, the University of Amsterdam, Amsterdam, Netherlands).

## Results

Participants performed 149 ± 27 DJs (range: 100–180). At POST, MVC decreased to 87.8 ± 10.5% of the PRE value (from 651 ± 152 N to 571 ± 140 N; *p* < 0.001; d = 1.2) and DB100 decreased to 80.6 ± 13.9% of the PRE value (from 257 ± 39 N to 208 ± 48 N; *p* < 0.001; d = 1.4). On the contrary, VA did not change significantly (94 ± 3% vs. 92 ± 3%; *p* = 0.14; d = 0.4).

**FIGURE 3:**
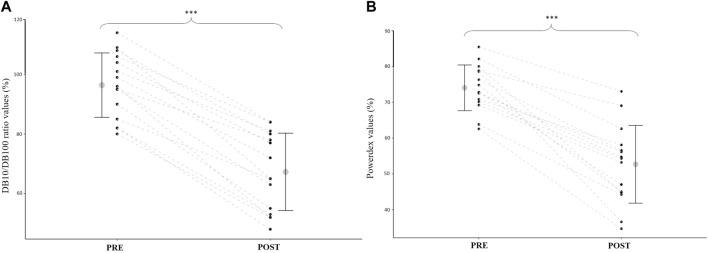
**(A)**: Quadriceps DB10/DB100 ratio before (PRE) and after (POST) the drop jump task. **(B)**: Powerdex values before (PRE) and after (POST) the drop jump task. Mean and standard deviation, as well as individual data (black points linked by dashed lines), are presented. *** = significantly different from PRE (*p* < 0.001).

**FIGURE 4:**
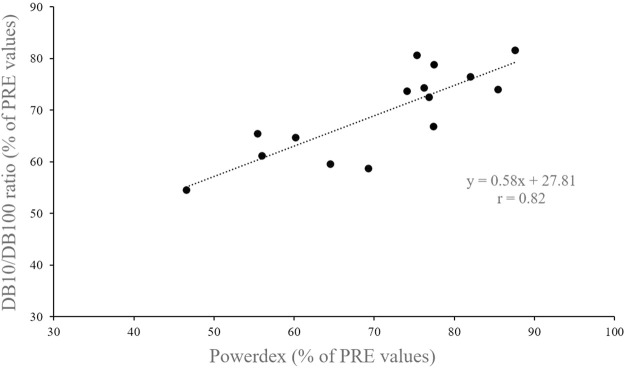
Relationship between Powerdex and DB10/DB100 ratio values recorded after the drop jump task (i.e., in POST).

At POST, Powerdex fell to 71.0 ± 12.0% of the initial value (from 74.0 ± 6.4% to 55.7 ± 10.9% *p* < 0.001; d = 2.4; Figure 3A). The DB10/DB100 ratio fell to 69.5 ± 8.6% of the initial value at POST (from 96.4 ± 10.9% to 67.3 ± 13.0%; *p* < 0.001; d = 3.7; Figure 3B). There was no significant difference between POST values (expressed in percentage of PRE values) of the DB10/DB100 ratio and Powerdex (*p* = 0.44), and there was a significant correlation between those values (r = 0.82, *p* < 0.001; Figure 4).

## Discussion

The present study aimed to compare LFF measurement after an intense eccentric task obtained either using an on-field device or through classic in-lab procedures. The main findings are that 1) on-field and in-lab methods detected similar amount of LFF and 2) the amplitudes of LFF were correlated between the two methods.

In the present study, participants performed a series of DJs, ranging from 100 to 180 jumps. Such exercises consisted of stretch-shortening cycles (i.e., an eccentric contraction during the ground contact phase followed by a concentric contraction during propulsion), with a strong eccentric component. As a result, we observed a marked maximal force depression in POST, which is indicative of performance fatigability, as already reported in the literature after a similar exercise (Kamandulis et al., 2019). As previously reported by others (Kamandulis et al., 2010), this decrease in MVC was not associated with an alteration of the central drive (i.e., no change in VA). On the contrary, it was accompanied by an alteration of muscle contractility as suggested by the significant decrease in evoked forces on relaxed muscles. Because a 10-min rest period was provided before this measurement, most of the acute metabolic perturbations were probably gone, especially considering the nature of the task, that is, eccentric contractions with a low metabolic cost. Altogether, our results suggest that performance fatigability mainly originated within muscle fibers and was mostly independent of metabolic perturbations.

In the present study, a series of DJs resulted in evoked force depression which was more marked in response to low- (i.e., DB10) than high-frequency (i.e., DB100) nerve stimulation. Consequently, there was an alteration in the DB10/DB100 ratio at POST, suggesting the presence of LFF in the quadriceps. While it must be acknowledged that the use of doublets may underestimate the magnitude of LFF compared to low- and high-frequency tetanic stimulations (Ruggiero et al., 2019), it still allows a valid detection of LFF with limited discomfort for the participants, especially when using femoral nerve stimulation (Verges et al., 2009). Alternatively, evoked force to trains of muscle submaximal stimulation can be used as a less painful assessment method (Martin et al., 2004). Accordingly, several in-lab studies previously reported decreases in low- to high-frequency force responses to trains of quadriceps muscle stimulation applied at either submaximal (Skurvydas et al., 2002; Martin et al., 2004) or supramaximal (Kamandulis et al., 2010; Skurvydas et al., 2016; Kamandulis et al., 2017; Kamandulis et al., 2019; Muanjai et al., 2020) intensity after repeated DJs.

Similar to the DB10/DB100 ratio, the Powerdex obtained through the on-field device decreased after DJs. When comparing LFF evaluation through the on-field device with peripheral nerve electrical paired stimulation, no significant difference was found between POST values (expressed in percentage of PRE values). Moreover, values obtained with these two methods were highly correlated. These findings suggest that the on-field device can effectively evidence the presence of LFF, but also provide a valid measurement of LFF, at least when considering doublets as a reference in-lab method. These results are promising and suggest the on-field device could be an alternative method to in-lab technics for the measurement of LFF in the field.

As mentioned earlier, the causes of LFF after DJs in the present study are likely not associated with metabolic factors because of the 10-min resting period before POST measurements and the nature of the fatiguing task. Instead, mechanical factors probably contributed to the presence of LFF. For instance, eccentric contractions may lead to muscle damage that can alter connections between the sarcoplasmic reticulum and the T-tubules. More specifically, the coupling between the dihydropyridine receptor and the ryanodine receptor can be damaged (Balog, 2010), limiting the amount of Ca^2+^ released from the sarcoplasmic reticulum for each action potential. As a result, the amount of force that can be produced when the action potential comes into the fiber is decreased (Keeton and Binder-Macleod, 2006). This is notably more evident at low frequency than at high frequency due to the sigmoidal relationship between the Ca^2+^ concentration and the force output. Consequently, a small reduction in the Ca^2+^ concentration will have more effect on the steep part of the curve (corresponding to low frequency) than on the horizontal part (corresponding to high frequency) (Jones, 1996; Keeton and Binder-Macleod, 2006; Allen et al., 2008).

Because the on-field device can evidence the presence of those LFF consequences similar to the in-lab methods, this device could integrate the athletes’ routine in order to assess LFF in their quadriceps on a daily basis, especially considering that its use is very easy and short (i.e., 2 min for one leg), contrary to current existing in-lab methods. This on-field measurement has potential implications for athletes. For instance, LFF assessment with such an on-field device could be easily performed on both legs, allowing to highlight a possible imbalance in the decrease of their respective Powerdex, likely indicating a greater work on one leg than on the other one. Consequently, the results can help to optimize training with tailored exercises. The measurements can also be performed continuously throughout the season to evaluate the fatigue state of the athletes and thus adapt the volume or type of training to avoid injuries or overtraining.

In conclusion, while the assessment of LFF is generally restricted to laboratory settings, our results suggest that its assessment can also be performed on the field with dedicated devices such as the Myocene^®^ device we tested in the present study. For instance, similar results we obtained with the in-lab and on-field procedures suggest that the on-field device is a valid device to assess LFF after a strenuous eccentric exercise (i.e., repeated DJs in the present study). Using such a device could provide better accessibility and acceptability for the on-field assessment of muscle fatigability in athletes. It is thought that better monitoring of fatigue in athletes could help improve performance (i.e., through tailored training prescriptions) and may further decrease the risk of injury or overtraining syndrome.

## Data Availability

The raw data supporting the conclusions of this article will be made available by the authors, without undue reservation.
